# Development of a novel human triculture model of non-alcoholic fatty liver disease and identification of berberine as ameliorating steatosis, oxidative stress and fibrosis

**DOI:** 10.3389/fphar.2023.1234300

**Published:** 2023-10-19

**Authors:** Hossein Rafiei, Michelle Yeung, Sara Kowalski, Gerald Krystal, Ingrid Elisia

**Affiliations:** Terry Fox Laboratory, BC Cancer Research Institute, Vancouver, BC, Canada

**Keywords:** berberine, fibrosis, inflammation, NAFLD model, steatosis

## Abstract

**Objectives:** Non-alcoholic fatty liver disease (NAFLD) and its progression to non-alcoholic steatohepatitis (NASH) and hepatocarcinoma is a serious and growing problem. However, the development of new therapies is severely hindered by a lack of high-throughput assays for drug testing.

**Methods:** We have developed a simple transwell assay comprised of HepG2 hepatocytes, hepatic LX-2 stellate cells, and differentiated THP-1 cells. The cells were incubated with an activating mixture containing the NASH-associated risk factors, glucose, insulin, free fatty acids (FFAs), and lipopolysaccharide (LPS) for 72 h. We compared different combinations of culture conditions to obtain a model system that recapitulates the main features of NAFLD/NASH, i.e., increased steatosis, reactive oxygen species (ROS), secretion of pro-inflammatory cytokines/chemokines, and presence of fibrosis. To confirm the usefulness of the optimized model system, we screened for compounds that inhibit steatosis in the hepatocytes and evaluated the most effective compound in the triculture model system.

**Results:** The activating mixture stimulated HepG2 cells in this triculture to accumulate more fat and produce higher levels of reactive oxygen species (ROS) than HepG2 cells in monocultures. As well, higher levels of inflammatory cytokines and chemokines (IL-8, IL-6, MIP-1α, *etc.*) were produced in this triculture compared to monocultures. In addition, in all LX-2 monocultures and cocultures, exposure to the activating mixture increased markers of fibrosis. A major strength of our triculture system is that it makes possible the simultaneous monitoring of 4 main features of NASH, i.e., steatosis, oxidative stress, inflammation and fibrosis. Screening potential modulators that may reduce steatosis in HepG2 cells revealed the protective effects of the isoalkaloid, berberine. Tested using this novel triculture assay, treatment with 5 µM berberine decreased steatosis and ROS in HepG2 hepatocytes, reduced inflammatory cytokine production and inhibited collagen production from LX-2 cells.

**Conclusion:** This simple triculture model recapitulates the main features of NAFLD/NASH and should be useful for high-throughput preclinical drug discovery. In this model, berberine showed promising results in decreasing steatosis and ROS and protection against fibrosis.

## 1 Introduction

Non-alcoholic fatty liver disease (NAFLD) affects 24% of the general population, 75% of patients with type 2 diabetes and 95% of individuals with obesity (Medina et al., 2004). NAFLD patients exhibit a spectrum of manifestations that can progress from simple accumulation of fat (steatosis), to non-alcoholic steatohepatitis (NASH), fibrosis, cirrhosis, and hepatocellular carcinoma. While NAFLD is likely to become the leading cause of end-stage liver disease in the coming decades, there are currently no approved therapies for either NAFLD or NASH (Younossi et al., 2016; Younossi et al., 2018).

The underlying molecular mechanisms leading to the progression of NAFLD to NASH are complex and multiple factors may work in parallel in the disease process (Müller and Sturla, 2019). For example, hepatic lipid accumulation (steatosis) occurs due to increased hepatic *de novo* lipogenesis, increased uptake of circulating free fatty acids (FFAs), mainly originating from adipose tissue lipolysis, or from dietary fats (Donnelly et al., 2005). This increase in intracellular lipids, coupled with oxidative stress and inflammation, likely contributes to the development of NASH (Feaver et al., 2016). As well, quiescent hepatic stellate cells (HSCs) located in the space of Disse, play a key role in the development of hepatic fibrosis and cirrhosis (Lotersztajn et al., 2005). Following liver injury, these quiescent HSCs become activated and differentiate into highly proliferative myofibroblast-like cells which release inflammatory and fibrogenic mediators that cause extracellular matrix accumulation within the liver microenvironment, leading to fibrosis (Loreal et al., 1993; Hernandez-Gea and Friedman, 2011). In addition to the hepatocytes and the HSCs, activation of liver-resident macrophages (Kupffer cells), with their consequent production of cytokines and chemokines, contributes to NASH by promoting inflammation which, in turn, stimulates collagen production by HSCs (Marra and Lotersztajn, 2013).

While many potential therapeutics for NAFLD are currently in clinical trials, the complexity of the disease and lack of mechanistic clarity regarding its key drivers (Feaver et al., 2016) make progress difficult. In order to find new drug targets, a number of animal and cell models have been developed (Cole et al., 2018). However, animal models are not a practical approach for high throughput drug discovery. Thus, to both study the underlying mechanisms, and especially to screen for new therapeutic drugs for NAFLD/NASH, it is crucial to develop a simple, reliable *in vitro* model that displays the main features of NASH.

Most *in vitro* studies looking into the molecular mechanisms that lead to NAFLD have used monocultures involving either primary mouse or human hepatocytes (Akazawa et al., 2010; Sharma et al., 2011) or immortalized human hepatocyte cell lines such as HepG2, Huh7, and HepaRG (Anthérieu et al., 2011; García-Ruiz et al., 2015; Michaut et al., 2016; Rafiei et al., 2019a). Recent longitudinal studies, however, suggest that the presence of fibrosis is a more important factor in predicting outcomes of patients with NAFLD than other histologic features (Angulo et al., 2015), highlighting the need to measure fibrosis as a key feature to mimic NASH/NAFLD. A monoculture system that only measures steatosis in hepatocytes therefore is likely insufficient to recapitulate the pathogenesis of NASH/NAFLD. To address this shortcoming, several *in vitro* cell models have been used to study fibrogenesis in NASH, including primary rat and human hepatic stellate cells (Bates et al., 2020), and the human stellate cell lines, LX-1 and LX-2. Moreover, a few studies have recently used cocultures of the human hepatocyte cell line, Huh7, and the hepatic stellate cell line LX-2 to study the crosstalk between these cells in disease progression (Barbero-Becerra et al., 2015). Interestingly, such coculture models demonstrated the critical role of hepatocyte-HSC interplay in the activation of HSCs, a main feature of NASH. Co-culture of HepG2 hepatocytes with THP-1 monocytes, differentiated into macrophages, has also demonstrated the role of inflammatory cytokines in NAFLD progression to NASH (Chen and Ma, 2019). These studies have suggested that such co-culture models better mimic the main features of NAFLD/NASH, i.e., steatosis, inflammatory responses and fibrogenesis, than monoculture models (Barbero-Becerra et al., 2015). However, since it is likely that the development of NAFLD/NASH is a result of the interplay amongst the three major liver-resident cells (hepatocytes, HSCs and activated macrophages), a model system containing representatives of all three cell types would be desirable (Friedman, 2008; Barbero-Becerra et al., 2015; Sanches et al., 2015). In the present study we have developed a triculture model consisting of HepG2, LX-2 and differentiated THP-1 cells and compared the results obtained with this model to results with mono and dicultures following exposure to an activating mixture.

We then used this triculture model to screen for compounds that might protect against NAFLD/NASH. From the list of compounds tested, we identified berberine as a promising agent that could decrease steatosis. Berberine, an isoquinoline alkaloid isolated from plants such as *Berberis vulgaris* (barberry), *Berberis aristata* (tree turmeric) and *Mahonia aquifolium* (Oregon grape), has been used for more than 3,000 years in traditional Chinese medicine (Zu Zhu et al., 2004). *Berberis vulgaris* grows in vast areas of Asia, America, and Europe and it is well known in Iran because its dried fruits are widely used as a food additive in culinary dishes (Fouladi, 2012). Berberine is also sold in pharmacies and stores as over-the-counter supplements (Marcheluzzo et al., 2021). Berberine has been shown to possess anti-inflammatory and anti-oxidative effects (Zhou et al., 2008; Jeong et al., 2009; Rafiei et al., 2017; Rafie et al., 2018), with reported efficacy in preventing the progression of NAFLD in preclinical (Sun et al., 2009; Zhang et al., 2016; Luo et al., 2019) and clinical studies (Yan et al., 2015; Wei et al., 2016) by modulating multiple metabolic mechanisms and lowering inflammatory responses (Zhu et al., 2016). Hence, berberine was tested in our triculture model to further validate the triculture system.

## 2 Materials and methods

### 2.1 Cell culture conditions and cell ratios

**FIGURE 1:**
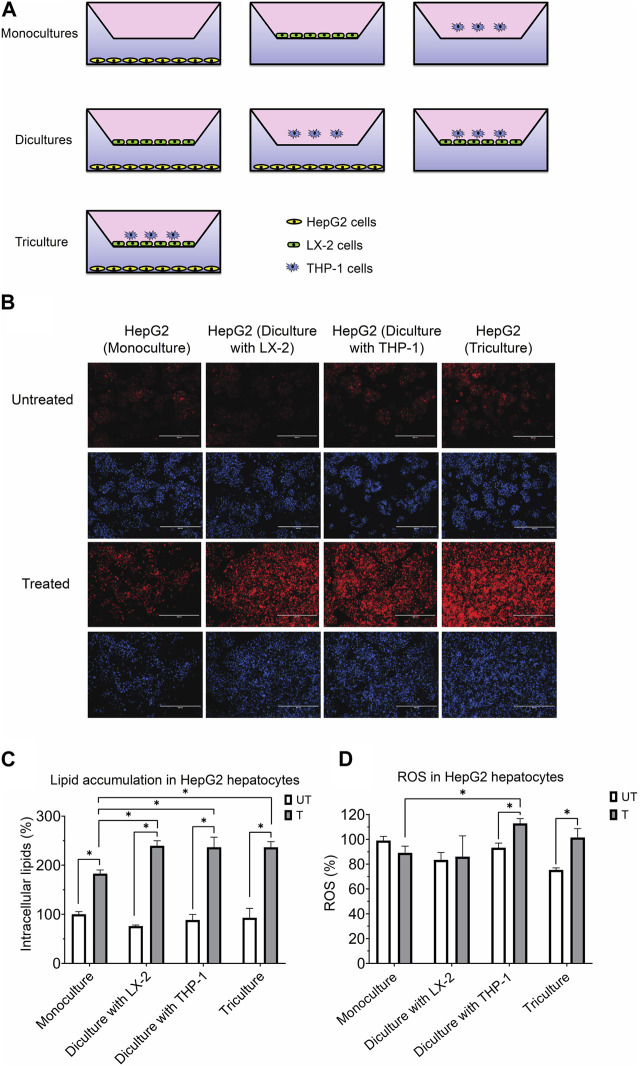
**(A)** Experimental design for the coculture models using the transwell system. **(B)** Representative photomicrographs (original magnification, ×20) of hepatocytes treated with or without the activating mixture and stained with Nile Red and Hoechst are shown. **(C)** Comparison of the effect of treatment with activating mixture for 72 h on HepG2 cells lipid accumulation in mono, di, and triculture condition. Intracellular lipids after quantification of Nile Red fluorescence using a microplate reader. Nile Red readings were normalized to fluorescence of nucleus-staining dye, Hoechst, which was used to control for cell number and proliferation. **(D)** Comparison of the effect of treatment with activating mixture for 72 h on ROS generation in HepG2 cells in mono, di, and triculture condition. Intracellular ROS in HepG2 cells after quantification of DCFH-DA fluorescence using a microplate reader. DCFH-DA readings were normalized to Hoechst fluorescence. In the graphs, untreated HepG2 monoculture reading was set at 100% and data are presented as % of the untreated HepG2 monoculture cells. * Significantly different at *p* < 0.05 with one-way ANOVA. UT, Untreated; T, Treated.

HepG2, LX-2 (a kind gift from Dr. Naglaa Shoukry at the Centre de Recherche du CHUM, Montreal, Quebec) and THP-1 cell lines were maintained in MEM containing 5.5 mM glucose, 2 mM glutamine with 10% fetal bovine serum (FBS) and 1% penicillin/streptomycin at 37^ο^C in a 5% CO_2_ incubator. HepG2 cells were seeded into the bottom compartment of a 24-well transwell (pore size 0.4 µm, Corning Inc. United States) and LX-2 into the top compartment, at 1.6 × 10^5^ cells each (Figure 1A). THP-1 cells were first differentiated to acquire a macrophage-like phenotype by exposure to 50 ng/mL phorbol 12-myristate 13-acetate (PMA) (Sigma, United States) for 24 h. These differentiated cells were then added to the top insert at 1.6 × 10^4^ cells. The final HepG2:LX-2:THP-1 cell ratio was 10:10:1. In preliminary experiments a HepG2:LX-2:THP-1 ratio of 10:1:1 was used to represent the physiological ratio of the cells in human liver and the results were similar, in terms of activating mixture-induced steatosis, ROS, inflammatory cytokines/chemokines, fibrosis and cell viability, to that obtained with the 10:10:1 ratio. Since the results from both triculture ratios were comparable, a ratio of 10:10:1 was used in order to obtain sufficient protein and RNA from LX-2 cells for Western blot and PCR assays, respectively.

### 2.2 Treatment with the activating mixture and berberine

To induce features associated with NAFLD/NASH *in vitro*, we added an activating mixture containing a final concentration of 11 mM glucose, 10 nM insulin, 100 µM oleic and 25 µM palmitic acid, 10 ng/mL lipopolysaccharide (LPS), and 3 ng/mL transforming growth factor beta (TGF-β) for 72 h to the cells. The concentrations of these components were based on differences in the plasma of healthy and NASH patients (Chitturi et al., 2002; De Almeida et al., 2002) and on earlier studies (Feaver et al., 2016), but with some modifications to account for cell toxicities in our model. We added LPS to the mixture since higher circulating levels of LPS, due to disruption of the intestinal barrier, have been implicated in insulin resistance and systemic inflammation, and associated with the development and progression of NAFLD (Ma et al., 2017; Bessone et al., 2019). Stocks of 120 mM oleic acid and 75 mM palmitic acid in ethanol were diluted in MEM containing 1% bovine serum albumin (BSA) to reach their final concentrations. For studies involving berberine, the cells were first incubated with berberine for 1 h prior to adding the activating mixture. At the end of the 72 h incubation period, cells were harvested for assays and the culture media frozen for further analyses.

### 2.3 Cell viability and proliferation assays

Prior to culturing the cells, THP-1 and LX-2 cells were first stained with carboxyfluorescein succinimidyl ester (CFSE) and CellTrace 670 (both from Thermofisher Scientific, Waltham, Massachusetts, USA), respectively, using the manufacturer’s protocols and then cocultured for 72 h HepG2 cells were not stained since they were seeded separately from LX-2 and THP-1 cells at the bottom of the transwell. In addition, all the cells were also stained with GhostDye Violet 450 viability dye (Tonbo Biosciences, San Diego, CA) to identify dead cells. Counting beads (CountBright™, Thermo Fisher) were added to each group of cells for quantification. Cell viability was determined by counting labeled cells using a BD LSR Fortessa flow cytometer (BD Biosciences). Data analysis was performed using FlowJo Software V10.3 (FlowJo, Ashland, OR). For the MTT assay, the culture media was replaced with MTT solution (final concentration 0.5 mg/mL) and the cells were incubated at 37°C for 4 h. After incubation, 10% sodium dodecyl sulfate was added, incubated overnight and the absorbance read with a plate reader at 570 nm.

### 2.4 Intracellular lipid measurement

Intracellular lipid was determined using Nile Red dye (MP Biomedicals, Santa Ana, CA, USA), as previously described (Rafiei et al., 2019b). Following treatment with the activating mixture for 72 h, HepG2 cells were washed with PBS and incubated with Hank’s balanced salt solution (HBSS) containing 1 μg/mL Nile Red and the cells were incubated in the dark for 30 min at 37°C. The cells were then carefully washed twice with phosphate buffered saline (PBS) and the fluorescence of each well read using a Synergy HT microplate reader (Bio Tek Instruments^®^, Synergy HT, Winooski, VT) at 488 nm excitation and 585 nm emission. The data were normalized to the nucleus-staining fluorescent dye, Hoechst (Thermofisher Scientific, Waltham, MA, USA), which was used to control for the number of cells and cell proliferation. Fluorescence images were captured using an EVOS fluorescent microscope.

### 2.5 Intracellular ROS measurement

Intracellular ROS was measured using fluorogenic probe 2′,7′-dichlorodihydrofluorescein diacetate (DCFH-DA), according to the manufacturer’s protocol (Molecular probes). After 72 h of incubation with the activating mixture, cells were washed and the medium replaced with fresh medium containing 25 µM DCFH-DA. After incubation with DCFH-DA for 30 min at 37°C, cells were carefully washed twice with PBS and fluorescence of each well determined using a Synergy HT microplate reader (BioTek Instruments^®^, Synergy HT, Winooski, VT) at excitation wavelength 485 nm and emission 528 nm. The readings were normalized to Hoechst fluorescence.

### 2.6 Measurement of inflammatory cytokines and chemokines

Inflammatory cytokines IL-6 and IL-8 were quantified in cell culture media using BD OptEIA™ human ELISA sets (BD Biosciences, San Diego, CA, USA). We also quantified the level of an additional 35 cytokines/chemokines in the cell culture supernatant using a 35-plex Luminex assay (cat # LHC6005M, Thermofisher Scientific, Ottawa, ON, Canada).

### 2.7 RT-qPCR

Gene expression was determined using RT-qPCR as previously described (Rafiei et al., 2017). Briefly, following treatment of the cells with the activating mixture for 72 h, cells were carefully washed 2–3 times with PBS and total RNA extracted using an RNAeasy Mini Kit (Qiagen) according to the manufacturer’s instructions. Reverse transcription of 2 µg of total RNA into cDNA was performed in 20 µL reactions using a VILO cDNA synthesis kit (Invitrogen, USA) and a Thermocycler (BioRad). All primers were synthesized by Integrated DNA Technologies Company (IDT, USA) (Supplementary Table S1). Polymerase chain reactions were performed using a PCR system (ABI 7300; Applied Biosystems) using a power SYBR green real-time PCR master mix (Applied Biosystems), according to the manufacturer’s protocol. Relative mRNA expression was determined by a comparative method (2^−ΔΔCT^) using GAPDH and RPL13A as reference genes. All results were normalized to the reference genes.

### 2.8 Western blotting

Following treatment of the cells in mono, di, and tricultures with the activating mixture for 72 h, LX-2 cells were trypsinized and washed twice with PBS. Total LX-2 cell protein was extracted using 4°C RIPA buffer (Millipore, MA, USA) supplemented with Halt protease inhibitor (Thermofisher Scientific) and incubated on ice for 30 min. After centrifugation at 18,000xg for 20 min, the protein content of the supernatants was quantified using a BCA protein assay. Equal amounts of protein were then mixed with loading buffer and denatured at 100°C for 1 min. Then, 70 µg of protein was loaded into each gel well, electrophoresed and proteins transferred to a 0.45 µm PVDF membrane. The membrane was blocked with Tris-buffered saline containing 0.1% Tween-20 (TBST) and 5% skim milk powder for 1 h at 23°C. The membrane was then incubated overnight at 4°C with rabbit primary monoclonal antibodies for collagen I (1:1,000) (Abcam, ab34710) and GAPDH (1:10,000) (Fitzgerald Industries, Acton, MA, USA). Membranes were then washed 3 times for 5 min each with TBST and incubated for 1 h with anti-rabbit secondary antibody (1:10,000) (Cell Signaling Technology, Denvers, MA). The results were normalized to GAPDH.

### 2.9 Statistical analysis

Results are expressed as the mean ± standard deviation (SD) and all experiments were performed in triplicate. Data were analyzed with GraphPad Prism (version 9). The difference between groups was analyzed with one-way ANOVA using Tukey’s *post hoc* tests or unpaired Student’s t-test when applicable. The level of significance was *p* < 0.05.

## 3 Results

### 3.1 Comparison of triculture with mono and diculture models

Shown in Figure 1A is the triculture model system that we developed as well as other monoculture and diculture models that we used in this study.

#### 3.1.1 Cell viability

Preliminary experiments were carried out to determine the optimal concentrations of compounds within the activating mixture, *i.e.*, those that significantly increased steatosis, inflammatory cytokines/chemokines, ROS and fibrosis, with minimal effects on cell viability. Initially, an activating mixture containing 150 µM final concentration of FFAs (100 µM oleic acid and 50 µM palmitic acid) was used but this negatively affected LX-2 and THP-1 cell viability (data not shown). Specifically, palmitic acid had a negative effect on cell viability and its concentration was therefore reduced from 50 μM to 25 µM in the final mixture (data not shown). Our results showed that the final activating mixture used in this study, which contained 11 mM glucose, 10 nM insulin, 100 µM oleic and 25 µM palmitic acid, 10 ng/mL LPS, and 3 ng/mL TGF-β, was not toxic to LX-2 or HepG2 cells in any mono, di, and triculture condition. However, it was slightly toxic (∼10%) to the differentiated THP-1 cells under diculture and triculture conditions (Supplementary Figure S1).

#### 3.1.2 Intracellular lipid accumulation

To assess the effect of our activating mixture on lipid accumulation (steatosis), which is a hallmark of NAFLD, intracellular lipids within HepG2 hepatocytes in the mono, di and tricultures were stained with Nile Red fluorescent dye. Fluorescence images, representative of intracellular lipids, and their corresponding images stained with nucleus-staining Hoechst dye are shown in Figure 1B. The images clearly show higher lipid content in treated vs. untreated HepG2 cells. Reading Nile Red fluorescence with a plate reader revealed that treated HepG2 cells in diculture and triculture conditions accumulated more fat than treated HepG2 cells in monocultures (+30% increase, all *p* < 0.0001). Specifically, the intracellular lipids in activating mixture-treated vs. untreated HepG2 cells in the triculture model was increased by ∼154% (*p* < 0.0001) while there was only ∼83% (*p* < 0.0001) increase in lipid content in HepG2 monocultures following activating mixture treatment (Figure 1C).

#### 3.1.3 ROS

Increased oxidative stress and ROS generation has been shown to play a key role in the pathogenesis and progression of NAFLD to NASH (Takaki et al., 2013). We observed no change in intracellular ROS following activating mixture treatment of monocultures of HepG2 cells (*p* = 0.12) or when cultured together with LX-2 cells (*p* = 0.68). In contrast, incubation with the activating mixture for 72 h increased ROS content in HepG2 cells cultured with differentiated THP-1 cells in di and tricultures (all *p* ≤ 0.005) (Figure 1D). Our observation that ROS was only elevated in HepG2 cells when THP1 was present in the culture suggests that the presence of differentiated THP1 is required for ROS generation in HepG2 cells.

#### 3.1.4 Inflammatory cytokines and chemokines

**FIGURE 2:**
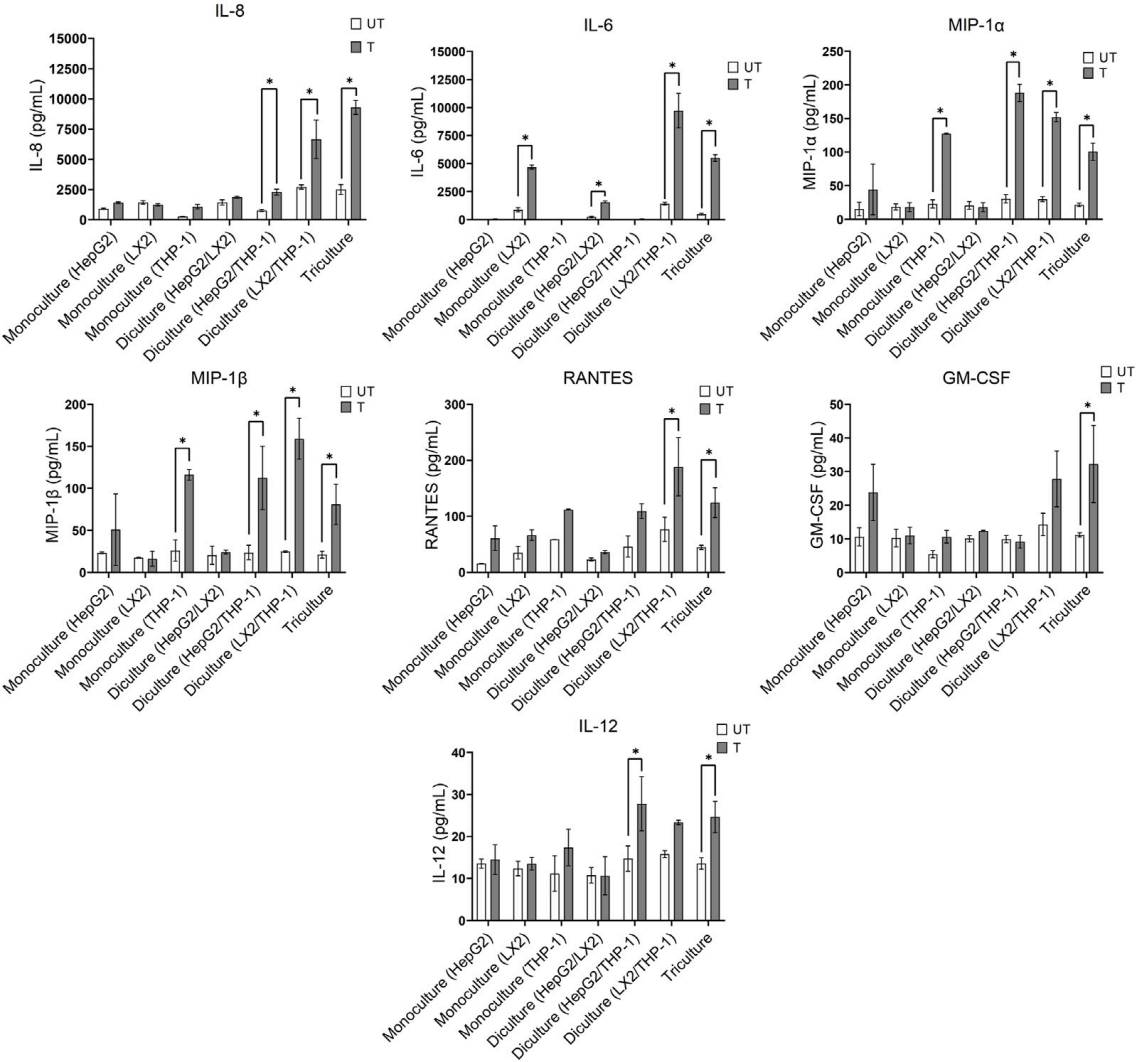
Effect of treatment with the activating mixture for 72 h on inflammatory cytokines and chemokines in the culture media collected from mono, di, and triculture conditions. Concentrations of cytokines/chemokines were determined using ELISA and 35-plex Luminex. * Significantly different at *p* < 0.05. UT, Untreated; T, Treated.

Oxidative stress triggers the production of inflammatory cytokines and chemokines, leading to inflammation, fibrogenic responses and the progression of NAFLD to NASH (Rolo et al., 2012; Takaki et al., 2013). We therefore compared the effect of a 72 h exposure to the activating mixture on cytokine and chemokine secretion in the mono, di and tricultures (Figure 2). Of the 35 inflammatory cytokines and chemokines tested, higher levels of interleukin 8 (IL-8), IL-6, macrophage inflammatory protein-1 alpha (MIP-1α), MIP-1β, RANTES, granulocyte macrophage colony-stimulating factor (GM-CSF), and IL-12 were observed in the culture media collected from activating mixture-treated tricultures. However, none of these cytokines/chemokines were elevated when only HepG2 monocultures were treated with the activating mixture (Figure 2). Importantly, we found that the interplay amongst the 3 cell types is important for a robust inflammatory response. For instance, differentiated THP-1 were likely the main cells producing IL-8 since there was a dramatic increase in IL-8 whenever differentiated THP-1 cells were co-incubated with LX-2 and HepG2 cells. However, the highest level of IL-8 was found in triculture conditioned media, indicating that the presence of both HepG2 and LX2 cells increased IL-8 production from THP-1 cells. Surprisingly, LX-2 cells appeared to be the primary IL-6 producing cells and dicultures with THP-1 dramatically increased IL-6 levels. Interestingly, dicultures of LX-2 cells with HepG2 cells reduced IL-6 levels compared to LX-2 monocultures, perhaps due to higher uptake of FFAs in the activating mixture by HepG2 cells and consequently less exposure of LX-2 cells to FFAs.

The activating mixture had no effect on other inflammatory cytokines and chemokines including tumour necrosis factor *a* (TNF-α), interleukin 1 alpha (IL-1α), IL-17F, IL2R, IFN-γ, IL-10, and IL-1β in the 35-plex Luminex assay (Supplementary Figure S2).

#### 3.1.5 Fibrogenesis

**FIGURE 3:**
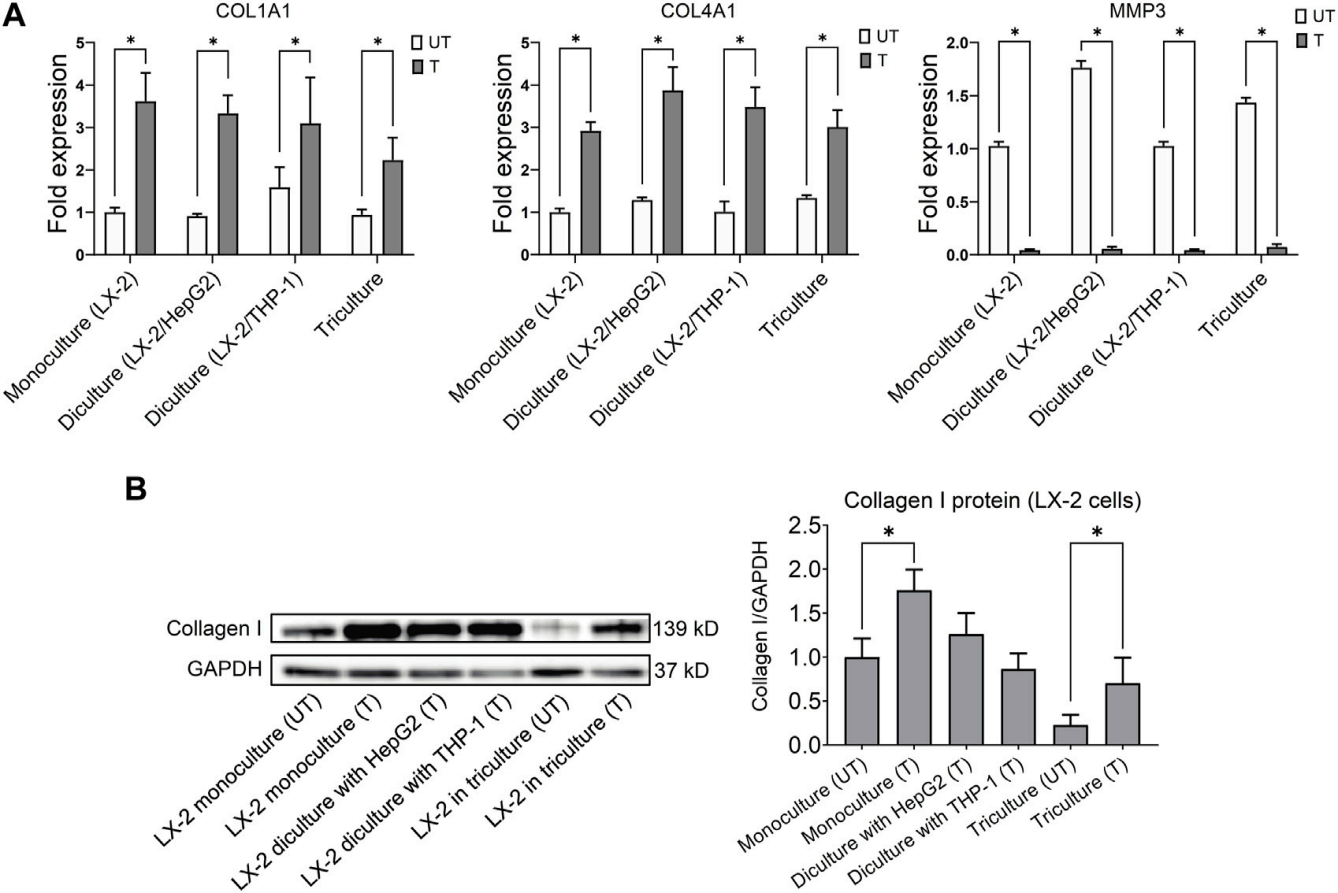
**(A)** Comparison of the effect of treatment with the activating mixture for 72 h on the expression of genes involved in fibrosis in LX-2 cells in mono, di, and tricultures. **(B)** Effect of treatment with the activating mixture for 72 h on collagen I protein (a key marker of fibrosis) in LX-2 cells in mono, di, and triculture setting. * Significantly different at *p* < 0.05 with one-way ANOVA. UT, Untreated; T, Treated.

Activation of hepatic stellate cells following lipotoxicity and liver injury plays a key role in fibrosis, a hallmark of NASH (Bessone et al., 2019). We therefore investigated the effect of the activating mixture on collagen synthesis in LX-2 cells, which are widely used as an *in vitro* model of fibrogenesis. The expression of pro-fibrogenic gene collagen type I alpha 1 (COL1A1) and collagen type IV alpha 1 (COL4A1) mRNAs increased in activating mixture-treated vs. untreated LX-2 cells in mono, di, and triculture conditions. At the same time, the expression of anti-fibrogenic gene matrix metalloproteinase-3 (MMP3) was completely inhibited by treatment (all *p* ≤ 0.009) (Figure 3A). In general, treated LX-2 cells in di and tricultures showed a similar extent of fibrogenesis to that of treated LX-2 monocultures. To further confirm the effect of the activating mixture on fibrosis in LX-2 cells under different culture conditions, collagen I protein, a key marker of fibrosis, was measured by Western blots. As shown in Figure 3B, activating mixture exposure for 72 h increased collagen I protein in LX-2 cells in mono, di, and triculture conditions. Interestingly, collagen I protein in activating mixture-treated LX-2 monocultures was higher than that of treated LX-2 cells in tricultures, perhaps because the HepG2 cells were sequestering the components of the activating mixture, resulting in a lower concentration of activating mixture available to LX-2 cells. However, complicating this interpretation, even untreated LX-2 cells in tricultures showed lower baseline collagen I protein than that of untreated LX-2 cells in monocultures, perhaps suggesting the HepG2 cells were secreting a factor(s) that was inhibiting collagen formation in LX-2 cells. Another possibility is that, since high glucose concentration has been shown to stimulate type I collagen production by HSCs (Sugimoto et al., 2005), higher collagen I production in LX-2 monocultures might be due to there being a higher glucose to cell ratio in monocultures.

### 3.2 Protective effects of berberine in the triculture model

Since our activating mixture-treated triculture system recapitulated the main features of NAFLD/NASH and allowed for the simultaneous measurement of steatosis, oxidative stress, inflammation and fibrosis, we then used it to screen a number of nutritional and therapeutic compounds that might decrease one or more of these hallmarks of NAFLD/NASH. The compounds were first tested in HepG2 monocultures to identify those that reduced steatosis and those that were promising were further tested in tricultures. Amongst the compounds tested were dietary polyphenols and flavonoids, water and lipid-soluble vitamins, short-chain fatty acids, and lipid and glucose lowering medications such as metformin and pioglitazone (Table 1). Of the compounds screened, berberine showed consistently promising results in lowering intracellular lipids in HepG2 hepatocytes in both mono and tricultures. We therefore chose to further test the efficacy of berberine on the other main features of NAFLD/NASH with our triculture system.

**TABLE 1:** Percent reduction in activating mixture-induced steatosis in HepG2 monocultures by candidate compounds.

Candidate compound	Intracellular lipid (% control)
Short-chain fatty acids	
2 mM Ketone Ester	94
1 mM Sodium Acetate	95
0.5 mM Sodium butyrate	95
1 mM Sodium butyrate	98
2 mM Sodium butyrate	96^†^
0.5 mM Sodium propionate	77*
1 mM Sodium propionate	90
2 mM Sodium propionate	75*^†^
0.5 mM Sodium hydroxy butyrate	78*
1 mM Sodium hydroxy butyrate	83*
2 mM Sodium hydroxy butyrate	77*
0.5 mM 3 hydroxy butyrate	79*
1 mM 3 hydroxy butyrate	75*
2 mM 3 hydroxy butyrate	82*
Polyphenols, flavonoids, and herbal extracts/compounds	
1 µM Berberine	90
2.5 µM Berberine	91
5 µM Berberine	67*
10 µM Berberine	68*^†^
20 µM Berberine	68*^†^
5 µM Quercetin	88
10 µM Quercetin	83
1 µM Curcumin	90
3 µM Curcumin	85*
10 µM Curcumin	85^†^
20 µM Curcumin	85^†^
5 µM Resveratrol	89
10 µM Resveratrol	96
20 µM Resveratrol	94
10 nM Triptolide +5 µM Quercetin	81*^†^
30 nM Triptolide +10 µM Quercetin	83*^†^
0.4 mg/mL Green Tea extract	73*^†^
0.8 mg/mL Green Tea extract	45*^†^
10 nM Triptolide	78*^†^
30 nM Triptolide	75*^†^
50 nM Triptolide	86^†^
100 nM Triptolide	86^†^
Phenolic acids	
10 µM Protocatechuic acid (PCA)	99
20 µM PCA	99
10 µM 2,4,6-Trihydroxybenzaldehyde (THB)	88
20 µM THB	88^†^
Vitamins	
200 nM Vitamin D_2_	91
5 nM Vitamin D_3_	91
10 nM Vitamin D_3_	87
20 µM γ Tocopherol	85
100 µM γ Tocopherol	78*
100 µM Vitamin C	99
50 µM DHA	88
100 µM DHA	93
50 µM EPA	86^†^
100 µM EPA	95^†^
Therapeutic agents	
0.5 mM Metformin	85
2 mM Metformin	86
40 µM Pioglitazone	79*
0.5 nM Dexamethasone	95
Other compounds	
1 mM AICAR	85^†^

* Denotes a significantly (*p* < 0.05) reduced HepG2 intracellular lipid content relative to HepG2 cells exposed to the activating mixture for 72 h (control).

^†^ Indicates significant (*p* < 0.05) cytotoxicity as determined by MTT assay.

Results are expressed as the average of three replicates of a representative experiment.

The typical coefficient of variation was below 13%.

PCA, Protocatechuic acid; THB, 2,4,6-Trihydroxybenzaldehyde; EPA, Eicosapentaenoic acid; DHA, Docosahexaenoic acid.

#### 3.2.1 Cell viability of berberine-treated cells

As already mentioned, treatment with the activating mixture for 72 h did not affect viability of HepG2 and LX-2 cells but slightly (∼10%) decreased the viability of differentiated THP-1 cells. Dose response studies with berberine (0.1–20 µM) in HepG2 monocultures revealed that berberine at 5 µM was effective in significantly reducing HepG2 cell lipid accumulation with no toxicity. Since higher concentrations of berberine were toxic to the HepG2 cells, 5 µM berberine was chosen for further testing in the triculture model. In the triculture, while co-incubation with 5 µM berberine did not decrease viability of LX-2 and THP-1 cells, it slightly (∼8%) decreased the viability of HepG2 cells (Supplementary Figure S3).

#### 3.2.2 Effect of berberine on intracellular lipid accumulation and ROS

**FIGURE 4:**
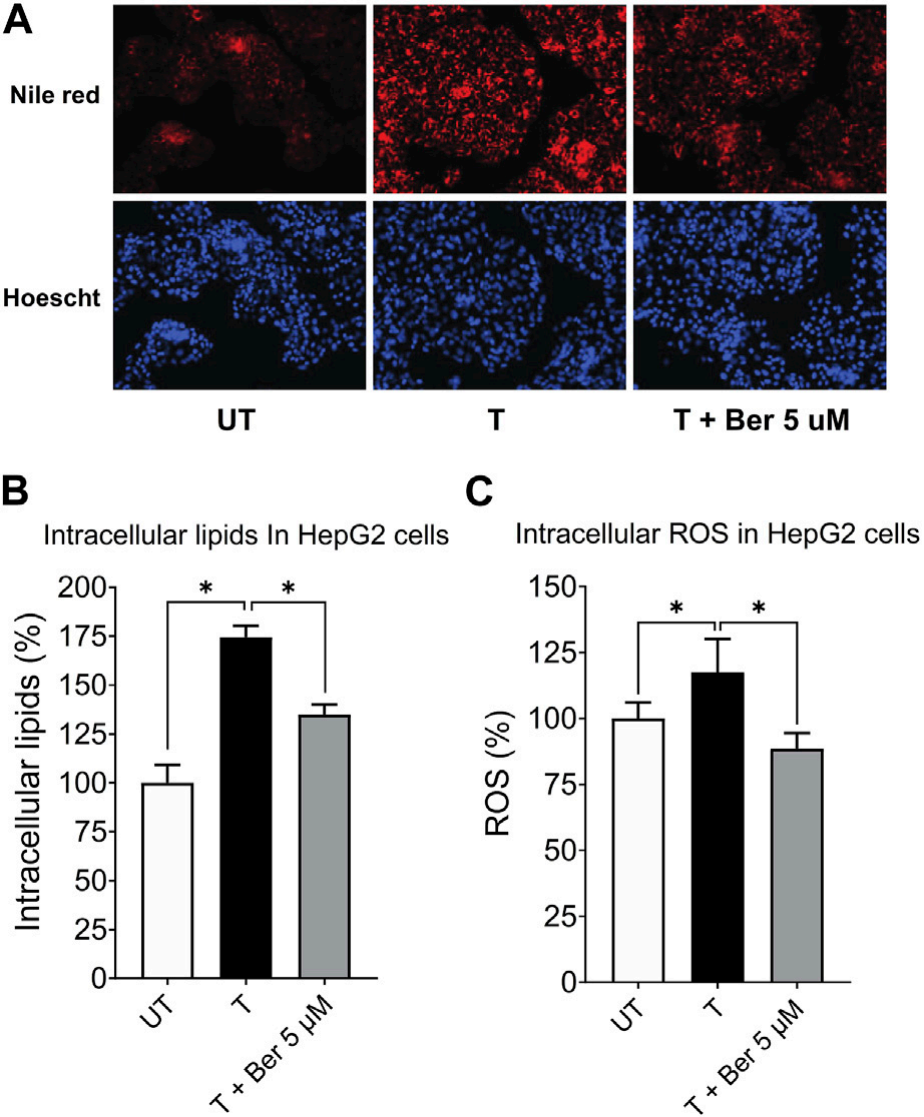
Protective effect of 5 µM berberine on intracellular lipid accumulation and ROS in HepG2 hepatocytes in the tricultures exposed to the activating mixture for 72 h. **(A)** Representative fluorescence images with a ×20 objective lens using Nile Red fluorescence probe showing intracellular lipid content. **(B)** Intracellular lipids in HepG2 hepatocytes in the triculture setting after quantification of Nile Red fluorescence using a microplate reader. Nile Red fluorescence was normalized to Hoechst fluorescence to control for cell proliferation. **(C)** Intracellular ROS in HepG2 cells in the triculture setting after quantification of DCFH-DA fluorescence using a microplate reader. DCFH-DA fluorescence was normalized to Hoechst fluorescence. Untreated was set at 100% and data are presented as % of the untreated cells. * Significantly different at *p* < 0.05 with one-way ANOVA. UT, Untreated; T, Treated; Ber, Berberine.

Representative fluorescence images of intracellular lipids in HepG2 cells stained with Nile Red, shown in Figure 4A, revealed a lipid-lowering effect of berberine. Treatment with the activating mixture for 72 h increased intracellular lipids (*p* < 0.0001) and ROS (*p* = 0.047) in HepG2 cells in the triculture model while co-incubation with 5 µM berberine protected against these increases (both *p* ≤ 0.006) (Figures 4B, C). To account for the effect of the activating mixture and berberine on cell viability and proliferation, Nile Red and DCFH-DA data were normalized to Hoechst levels. Of interest, berberine reduced ROS levels below that observed with untreated tricultures.

#### 3.2.3 Effect of berberine on inflammatory cytokines and chemokines

**FIGURE 5:**
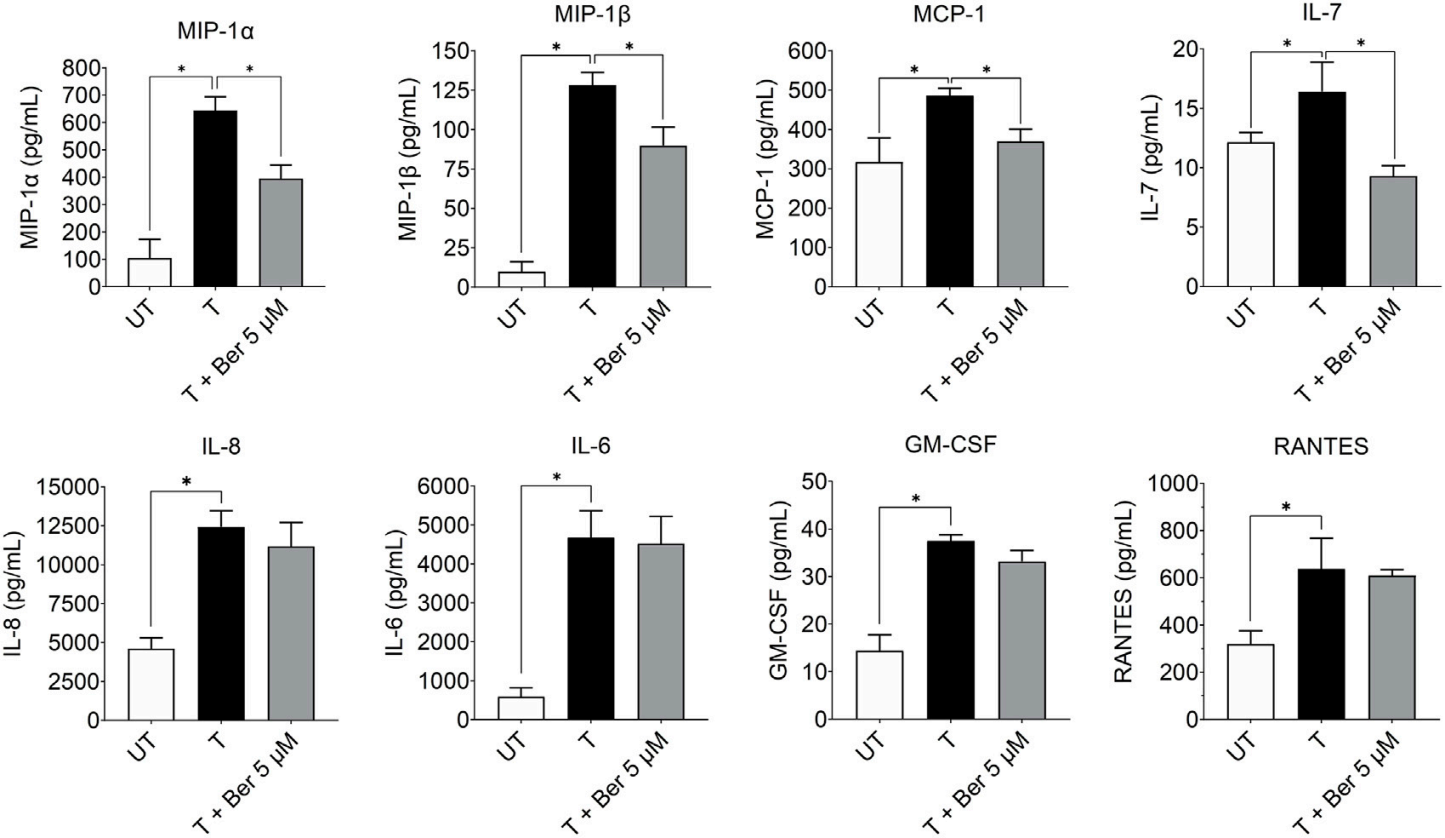
Effect of treatment with the activating mixture and 5 µM berberine for 72 h on pro-inflammatory cytokines and chemokines in culture media from tricultures. Concentrations of cytokines and chemokines were measured with 35-plex Luminex, except for IL-8 and IL-6, which were measured with ELISA. * Significantly different at *p* < 0.05 with one-way ANOVA. UT, Untreated; T, Treated; Ber, Berberine.

While treatment with the activating mixture for 72 h increased a number of proinflammatory cytokines and chemokines in the triculture model, co-incubation with 5 µM berberine only lowered MIP-1α (*p* = 0.004), MIP1β (*p* = 0.009), MCP-1 (*p* = 0.03), and IL-7 (*p* = 0.003) (Figure 5). Of interest, berberine reduced IL-7 levels below that observed with untreated tricultures. Berberine had no effect on IL-6, IL-8, RANTES and most cytokines and chemokines in the 35-plex Luminex panel (Figure 5; Supplementary Figure S4).

#### 3.2.4 Berberine and fibrogenesis

**FIGURE 6:**
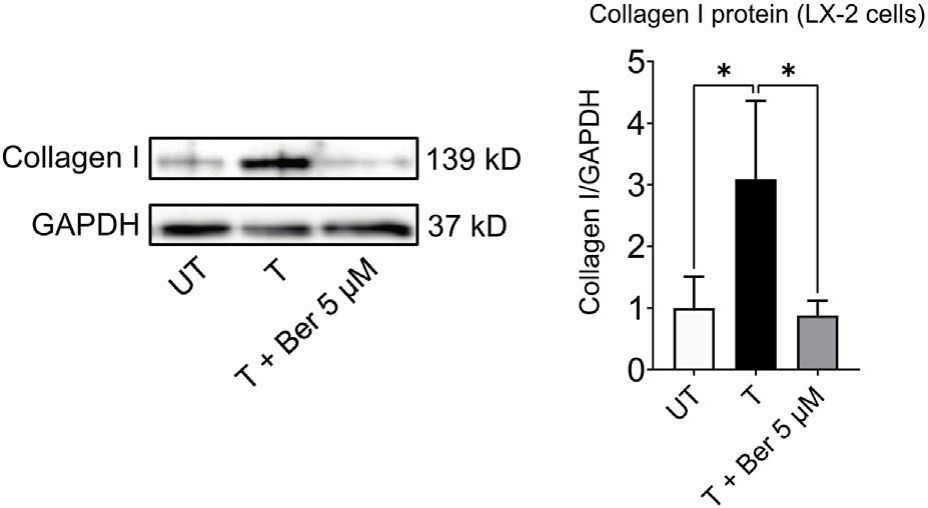
Berberine reduces activating mixture-induced collagen I protein levels in LX-2 cells in tricultures after 72 h * Significantly different at *p* < 0.05 with one-way ANOVA. UT, Untreated; T, Treated; Ber, Berberine.

While treatment with the activating mixture increased collagen I protein (*p* = 0.043), which is a hallmark of fibrogenesis in LX-2 cells in the triculture model, co-incubation with 5 µM berberine completely prevented this increase (*p* = 0.034) (Figure 6).

## 4 Discussion

Although the prevalence of NAFLD is high and increasing, in large part because of the obesity epidemic, there are currently no effective treatments and a dearth of investigational models. For progress to occur, more human-relevant systems that recapitulate the main features of NAFLD/NASH are needed for preclinical studies (Feaver et al., 2016). Studies to date have primarily used monocultures of either hepatocytic cells or hepatic stellate cells (Xu et al., 2005; García-Ruiz et al., 2015; Rafie et al., 2018). Dicultures of hepatocytes with hepatic stellate cells or macrophages have also been reported (Barbero-Becerra et al., 2015; Chen and Ma, 2019). For example, a recent NASH study in which the human hepatocyte cell line, Huh7, was cocultured with the human stellate cell line, LX2, demonstrated that this model retained key features of cytokine signaling and fibrosis (Barbero-Becerra et al., 2015). Specifically, when Huh7 and LX-2 cells were cocultured and stimulated with FFA for 24–144 h, activation of LX-2 cells, as evidenced by alpha-smooth muscle actin expression, was greater than when the LX-2 cells were cultured alone. The authors concluded that cell-to-cell contact between hepatocytes and stellate cells was important for the initiation of fibrogenesis (Barbero-Becerra et al., 2015). However, given that hepatocytes and hepatic stellate cells are separated by a membrane in our model, and we still observed an increase in fibrosis, suggests that flow of media between the 2 cell types might be sufficient for the activation of stellate cells.

Since macrophages have also been shown to play a role in NAFLD and progression to NASH, an *in vitro* model that incorporates hepatocytes, hepatic stellate cells and macrophages may best recapitulate NAFLD progression to NASH. However, only a limited number of studies have explored tricultures as an *in vitro* human liver system to mimic the features of NASH (Feaver et al., 2016; Suurmond et al., 2019). For example, Feaver et al. designed a transwell system in which primary human hepatocytes were cultured at the bottom and primary hepatic stellate cells and macrophages (MΦs) were cocultured at the top and perfused with an activating milieu containing glucose, insulin, FFAs, and inflammatory cytokines under hemodynamic flow to simulate NASH. Interestingly, the authors found that significant pathway correlations existed between their *in vitro* model and NASH patient biopsies, and that the activating milieu activated similar biological signatures in this system to that observed in NASH patients (Feaver et al., 2016). Our results are in line with this triculture study in which both their and our activating mixture increased steatosis in hepatocytes, elevated inflammatory cytokines and chemokines including IL-6, IL-8 and MCP-1, and increased fibrogenic activation markers such as collagen in hepatic stellate cells. However, their system, which relies on primary human liver samples and employs hepatic sinusoidal flow, is both not easily accessible and relatively difficult to set up, compared to our model. In another study in which HepG2 (or HepaRG) cells, human umbilical vein endothelial cells (HUVECs), and mouse Kupffer cells were cocultured as spheroids, the authors found the addition of hepatic Kupffer cells was necessary for robust lipid accumulation and ROS when the cells were exposed to steatosis-inducing media (containing palmitic and oleic acids) for 6–8 days (Suurmond et al., 2019). This is in agreement with our study in which coculturing differentiated THP-1 cells with HepG2 cells further increased lipid accumulation and ROS in HepG2 cells, compared to HepG2 cells in monocultures.

The aim of our work was to establish a simple and reliable triculture model using the three major liver-resident cells known to play key roles in the progression of NAFLD to NASH (Paschos and Paletas, 2009). Consistent with this, we found that when these tricultures were exposed to a physiologically relevant activating mixture they displayed some of the main features of NAFLD/NASH pathogenicity, i.e., increased steatosis, ROS, inflammatory cytokines/chemokines and fibrosis. Our activating mixture, unlike those used in many *in vitro* studies in which liver cells are treated with a single compound (i.e., FFA or glucose) at very high, non-physiological concentrations (LeCluyse et al., 2012), contained components that likely contribute to the activating state of NAFLD/NASH in humans and were used at near-physiological levels. Our results showed that HepG2 cells in triculture and diculture conditions accumulate more fat and generate more ROS than when cultured in monocultures. Comparison of mono, di, and triculture models in our study demonstrated that treatment with our activating mixture was unable to increase ROS in HepG2 cell monocultures but could increase ROS in the presence of differentiated THP-1 cells. Although previous studies have shown that incubation of HepG2 hepatocytes in monocultures with high concentrations of oleic and palmitic acid could increase ROS generation (Zhang et al., 2019; Sun et al., 2021), the concentrations of FFAs used were much higher than used in our study. Also, our hepatocytes and HSCs are separated by a membrane so they can easily be harvested independently and studied for molecular mechanisms underlying NAFLD/NASH.

Following screening with our novel triculture system of a large number of compounds, we identified berberine as one of the most promising candidates to ameliorate NAFLD. Specifically, we found that 5 µM berberine not only decreased lipid accumulation in HepG2 cells but ROS generation and collagen formation from LX-2 cells. As well, berberine decreased several activating mixture-induced inflammatory cytokines and chemokines, such as MIP-1α, MIP-1β, MCP-1, and IL-7 but did not protect against many others. Since MIP-1α, MIP-1β, MCP-1 are known chemokines that recruit immune cells to inflammatory sites, berberine may work in the initial phase of NAFLD/NASH by preventing inflammatory cell infiltration into the liver. Our finding that berberine reduced MCP-1 is also consistent with a report by Cui et al., who showed that berberine dose-dependently inhibited the mRNA expression of MCP-1 in the iris-ciliary body of rats treated with LPS (Cui et al., 2007). As well, MCP-1 has been reported to activate the fibrogenic activity of hepatic stellate cells (Kobayashi et al., 2018). Since berberine prevented activating mixture-induced fibrosis, and since MCP-1 is most likely produced by the monocyte-derived macrophage cell line, THP-1, we propose that berberine may suppress MCP-1 production from THP-1 cells, and this in turn reduces MCP-1-induced fibrogenesis in LX-2 cells. In our study, as mentioned above, berberine reduced steatosis and oxidative stress, which, along with other factors, are important mediators in the development of NASH and liver fibrosis (Bessone et al., 2019). This is consistent with the reported efficacy of berberine in preventing fibrosis *in vitro* and in a NASH-HCC mouse model system (Zhang et al., 2016; Luo et al., 2019; Yi et al., 2021; Liu et al., 2022). Our finding of collagen synthesis prevention in LX-2 cells by berberine is aligned with *in vitro* and animal studies that have shown berberine could prevent the progression of hepatic steatosis to steatohepatitis and fibrosis by modulating lipid metabolism and decreasing lipogenesis (Zhang et al., 2016). Taken together, we demonstrate that berberine is effective in reducing all the factors that may promote fibrogenesis in the liver, highlighting the potential benefit of berberine in NASH/NAFLD.

One of the limitations of this triculture system is that even though this model provides a simple way to explore the molecular mechanisms of NAFLD/NASH, it does not accurately replicate the complex interactions between the liver and other tissues. The communication between adipose tissue and the liver, for example, likely plays a pivotal role in the initiation and progression of NAFLD to NASH that this model cannot effectively recapitulate. Future research directions to enhance the current model system may involve the use of primary cells instead of cell lines and the addition of visceral fat cells.

## 5 Conclusion

In conclusion, we demonstrate that our activating mixture-activated triculture system, composed of HepG2, LX-2 and differentiated THP-1 cells, simulates the major features of human NAFLD/NASH. We show that while most *in vitro* studies use hepatocytes or HSC monocultures to study NAFLD and NASH they fail to represent the main features of the disease and do not take into account the interplay amongst the major liver cell types. When exposed to a near-physiological activating mixture, our simple triculture model displays multiple key features of NAFLD and NASH without the complexity of 3D or organ on a chip models. Advantages of our *in vitro* triculture 2D model are that the same methodologies utilized in monocultures can be applied and, importantly, it makes possible high-throughput testing of potential therapeutics via the simultaneous assessment of steatosis, inflammatory cytokines/chemokines, ROS and fibrosis. In this triculture model, hepatocytes and HSCs can be independently harvested and studied for classic features of NAFLD and NASH, while addition of differentiated THP-1 allows for the exploration of the role of inflammatory responses in disease progression. Berberine tested in this model at a physiological dose showed promising results in decreasing steatosis and ROS and protection against fibrosis, in agreement with clinical studies. Therefore, this triculture model mimics human NAFLD and NASH and should be useful for preclinical drug discovery.

## Data Availability

The raw data supporting the conclusion of this article will be made available by the authors, without undue reservation.
