# Supplementary material for: Development of a novel human triculture model of non-alcoholic fatty liver disease and identification of berberine as ameliorating steatosis, oxidative stress and fibrosis
Source: Front Pharmacol. 2023 Oct 19;14:1234300. doi: 10.3389/fphar.2023.1234300 (PMC10620695; doi:10.3389/fphar.2023.1234300)
Supplement: Supplementary file 1 [file DataSheet1.docx]

***Supplementary Material***

| Gene | Species | Primer sequence Forward (5’–3’) | Primer sequence Reverse (5’–3’) | Cycles  (Ct values) |
| --- | --- | --- | --- | --- |
| COL1A1 | Human | CCG GCT CCT GCT CCT CTT AGC G | CGT TCT GTA CGC AGG TGA TTG GTG G | 21 |
| COL4A1 | Human | CCT GGC TTG AAA AAC AGC TC | CCC TGC TGA GGT CTG TGA AC | 24 |
| MMP3 | Human | GAC AAA GGA TAC AAC AGG GAC | TGA GTG AGT GAT AGA GTG GG | 33 |
| GAPDH | Human | ATG GGT GTG AAC CAT GAG AAG | GAG TCC TTC CAC GAT ACC AAA G | 18 |
| RPL13A | Human | CAT AGG AAG CTG GGA GCA AG | GCC CTC CAA TCA GTC TTC TG | 23 |

**Supplementary Table 1.** The sequences of primers used to measure the expression of genes of interest

**Supplementary Fig. 1.** Effect of treatment with the activating mixture for 72 h on the viability of HepG2, LX-2, and THP-1 cells in mono, di, and triculture conditions. The cells were stained with fluorescent dyes CFSE and CT670 and analyzed by a FACS. * Significantly different at *P* < 0.05. UT: Untreated, T: Treated.

**Supplementary Fig 2.** Effect of treatment with the activating mixture for 72 h on inflammatory cytokines and chemokines in the culture media collected from mono, di, and tricultures. Concentrations of cytokines/chemokines were determined using a 35-plex Luminex assay.

* Significantly different at *P* < 0.05. UT: Untreated, T: Treated.

**Supplementary Fig. 3.** Effect of treatment with activating mixture and 5 µM berberine for 72 h on viability of HepG2, LX-2 and THP-1 cells in the triculture model. The cells were stained with fluorescent dyes CFSE and CT670 and analyzed by FACS. * Significantly different at *P* < 0.05. UT: Untreated, T: Treated, Ber: Berberine.

**Supplementary Fig. 4.** Effect of treatment with the activating mixture and 5 µM berberine for 72 h on pro-inflammatory cytokines and chemokines in culture media from tricultures. Concentrations of cytokines and chemokines were measured with a 35-plex Luminex assay. *Significantly different at P < 0.05 with one-way ANOVA. UT: Untreated, T: Treated, Ber: Berberine.
